# The Novel LncRNA WASH5P Inhibits Colorectal Cancer Carcinogenesis *via* Targeting AKT Signaling Pathway

**DOI:** 10.3389/fonc.2022.923425

**Published:** 2022-07-11

**Authors:** Hongyun Wei, Tao Mao, Qian Zhang, Keyu Ren, Xingsi Qi, Yunmei Zhang, Bin Cao, Yanchun Jin, Zibin Tian, Linlin Ren

**Affiliations:** Department of Gastroenterology, Affiliated Hospital of Qingdao University, Qingdao, China

**Keywords:** LncRNA WASH5P, AKT signaling pathway, colorectal cancer, proliferation, cancer metastasis

## Abstract

Emerging evidence has shown that long non-coding RNAs (lncRNAs) play an important role in colorectal cancer (CRC) carcinogenesis, so more specific mechanisms of key lncRNAs in CRC initiation and development are needed. Here, we evaluated the expression profiles of lncRNAs in CRC tissues and identified a novel lncRNA generated from the pseudogene Wiskott-Aldrich syndrome protein (WASP) family homolog 5, termed lncRNA WASH5P. However, the role and potential molecular mechanism of this novel lncRNA in diseases, including CRC carcinogenesis, is unknown. Our present study found that WASH5P was significantly downregulated in CRC cell lines and tissues compared with normal controls. The ectopic expression of WASH5P in CRC cells could significantly inhibit CRC cell proliferation, invasion, and migration. In addition, WASH5P could increase the expression of E-cadherin and decrease Vimentin expression. WASH5P-overexpressing CRC cells developed tumors more slowly in different mouse models. Meanwhile, the overexpression of WASH5P could significantly inhibit AKT activation *via* suppressing AKT phosphorylation. The treatment of PI3K/AKT (phosphatidlinositol 3-kinase /protein kinase B) signaling agonist 740Y-P rescued WASH5P-reduced AKT phosphorylation and abolished the inhibitory effects of WASH5P on cell viability, migration, and invasion. Moreover, 740Y-P restored the WASH5P-induced downregulation of p-AKT and vimentin and the upregulation of E-cadherin *via* Western blot. In summary, our findings suggested that the novel lncRNA WASH5P might be a potential candidate biomarker and therapeutic target that could inhibit CRC by repressing the AKT signaling pathway.

## Introduction

Although substantial progress has been made in cancer prevention, diagnosis, and treatment recently, all kinds of cancer are still the leading causes of death according to cancer statistics in 2021 (1). For colorectal cancer (CRC), it is still the fourth leading cause of cancer-related death worldwide (2), more than a hundred thousand new cases and fifty thousand deaths were estimated in 2021 (1). Genetic, environmental, epigenetic, and microbiological factors have been considered as the causing factors of CRC (3). However, molecular pathogenesis is still not fully clarified. Additionally, the current treatment strategies did not show a satisfying effect. Therefore, it is of critical importance to identify new effective biomarkers and elucidate the underlying mechanisms involved in CRC carcinogenesis.

As a class of RNA molecules, long non-coding RNAs (lncRNAs) with more than 200 nucleotides are located in the nucleus or cytoplasm (4), and play key roles in gene regulation in various human diseases, such as cancers (5, 6). Emerging evidences demonstrated the important role of lncRNAs in the initiation and maintenance of solid carcinomas (7–9). Recent studies have indicated that many lncRNAs, including MALAT1 (metastasis-associated lung adenocarcinoma transcript1), GAS5 (growth arrest-specific 5), and FLANC (flamingo non-coding RNA), were involved in the development of CRC (10–12). In addition, more and more studies revealed the relationship between lncRNAs and key signnaling pathways (13), including the AKT signaling pathway. Yao et al. showed that lncRNA MALAT1 could enhance the radioresistance of CRC *via* the AKT/YAP axis (14). In addition, lncRNA SPRY4-IT1 has functional interactions with PI3K/AKT signaling in the carcinogenesis of CRC (15). However, more specific mechanisms of key lncRNAs in CRC initiation and development need to be further teased out.

In the current study, we evaluated the expression profiles of lncRNAs in CRC tissues and identified a new lncRNA generated from the pseudogene Wiskott-Aldrich syndrome protein family homolog 5, termed lncRNA WASH5P. However, the expression level and potential function of WASH5P have not been reported until now. Here, we found that lncRNA WASH5P was significantly downregulated in both CRC tissues and cell lines. The biological roles of lncRNA WASH5P in CRC were genetically assessed in both *in vitro* and *in vivo* models. Further analysis revealed that lncRNA WASH5P inhibits CRC *via* suppressing the AKT pathway. In summary, we identified a novel lncRNA, WASH5P, which might be a potential candidate biomarker and therapeutic target in CRC *via* targeting the AKT signaling pathway.

## MATERIALS and methods

### Chemical

740Y-P, an activator of the AKT/AKT signaling pathway, was obtained from Med Chem Express (Cat. No. HY-P0175). The agent was dissolved with DMSO (dimethyl sulphoxide) and used to activate the AKT/AKT pathway in CRC cells.

### Bioinformatic Analysis

In the TCGA (The Cancer Genome Atlas) database, 30 paired normal and CRC samples from GSE74602 were included for further analysis. The related RNA-seq data were extracted. The expression of WASH5P was verified in TCGA and GTEx (the Genotype-Tissue Expression) databases. Statistical analysis was performed using R (v.3.5.1). Graphpad prism was used for plotting. Differential gene expression was considered significant if |logFC|>1 and adj.P.Val<0.05.

### Cell lines and Cell Culture

Four human colorectal cancer cell lines SW480、HCT116、RKO、 and HT29 and one normal colon cell line (NCM460) were obtained from the Cell Bank of the Chinese Academy of Sciences (Shanghai, China). NCM460, SW480, and HT29 were maintained in an RPMI-1640 (Procell, PM150110 Wuhan, China) medium supplemented with 10% fetal bovine serum and 1% penicillin–streptomycin (NCM Biotech, Suzhou, China c125c5) at 37°C with 5% CO_2_. HCT116 was maintained in a Dulbeco’s modified Eagle (Procell, PM150210 Wuhan, China) medium and RKO in an MEM (Minimum Essential Medium) medium (Procell, PM150410 Wuhan, China). All cell lines were validated by short tandem repeat profiling.

### Nuclear and Cytoplasmic RNA Fraction Isolations

Nuclear and cytoplasmic RNA was isolated with a Cytoplasmic and Nuclear RNA Purification Kit (Norgen Biotek Corp, Ontario, Canada) according to the manufacturer’s instructions. The cells were added with lysis buffer J including β-mercaptoethanol and incubated on ice for 5 min. After centrifugation for 10 min with 14,000 g, the supernatant was collected as the cytoplasmic fraction and the precipitation as the nuclear fraction. Then, RNA was extracted.

### RNA Extraction and Quantitative PCR

Total cellular RNA was isolated using a TRIzol reagent (Cat. No. 15596026, Thermo Fisher Scientific, Shanghai, China), dissolved in RNA-free H_2_O and stored at −80°C. RNA (1 μg) was used to obtain high-quality (complementary deoxyribonucleic acid) cDNA using the reverse transcription kit (Genecopoeia, Guangzhou, China) according to the manufacturer’s protocols. The PCR system was used to amplify all transcripts using the iQ SYBR Green Supermix’ (1708882AP; Bio-Rad, Shanghai, China). The primer sequences were as follows: WASH5P (sense 5’- GCGGCTCGCAGGCAC-3’, antisense 5’-GGATGAAGGGCACGGCA-3’). qPCR was designed with the following conditions: predenaturation at 95°C for 2 min followed by 40 cycles of denaturation at 95°C for 15 s, annealing and elongation at 60°C for 30 s. Relative transcripts were calculated using the 2^-ΔΔCt^ method. All experiments were repeated in triplicate.

### Protein Extraction and Western Blot

The extracted proteins from tissues or cells were resolved by sodium dodecyl sulfate - polyacrylamide gel electrophoresis gradient gel (5% spacer gel and 10% or 15% separation gel). After electrophoresis, samples were transferred to a polyvinylidene fluoride membrane, blocked at room temperature for 1 h with 5% milk, and incubated with a primary antibody at 4˚C overnight. Primary antibodies include a rabbit antibody against vimentin (1:2,000; Proteintech, 10366-1-AP), E-cadherin rabbit polyclonal antibody (1:2,000; Proteintech, 20874-1-AP), GAPDH rabbit polyclonal antibody (1:6,000; Proteintech, 10494-1-AP), GAPDH (glyceraldehyde-3-phosphate dehydrogenase) mouse monoclonal antibody (1:6,000; Proteintech, 60004-1-Ig), p-AKT antibody (1:1000; Y011054, abm, Richmond, BC, Canada), and AKT (1:1,000; Y409094, abm, Richmond, BC, Canada). The membranes were washed with a buffer three times, incubated with an HRP-conjugated anti-rabbit IgG (1:10,000; #14708, CST, USA) or anti-mouse IgG (H+L) (1:10000; CST, #14709, Shanghai, China) at room temperature for 2 h, and visualized with an ECL (Enhanced Chemiluminescence) kit (NCM Biotech, China).

### Vector Construction and Cell Transduction

For the downregulation of WASH5P, the siRNA-1 (upstream: 5’-GATCCGCTTACAGACTCTCTGTTTAACTCGAGTTAAACAGAGAGTCTGTAAGCTTTTTG-3’; downstream: 5’-AATTCAAAAAGCTTACAGACTCTCTGTTTAACTCGAGTTAAACAGAGAGTCTGTAAGCG-3’) and siRNA-2(upstream: 5’-GATCCGCTTGGTGTTCGCAACTTTGACTCGAGTCAAAGTTGCGAACACCAAGCTTTTTG-3’; downstream: 5’-AATTCAAAAAGCTTGGTGTTCGCAACTTTGACTCGAGTCAAAGTTGCGAACACCAAGCG-3’) sequence synthesized by Huada Technology (Shanghai, China) were cloned into the pLVX (pLVX-shRNA2-puro). In addition, a lentiviral plasmid containing the full length of WASH5P was constructed. The primer used for the overexpression of WASH5P is shown below: forward Primer 5’-GGATCTATTTCCGGTGAATTCGGAAGCGGCGGCGGGAGC-3’, reverse primer: 5’-CGCTCTAGAACTAGTCTCGAGGGCAGGAGGAGGGTGTGG-3’.

WASH5P-overexpressing or WASH5P shRNA (short hairpin RNA) lentivirus was produced in HEK293T cells, respectively. HCT116, SW480, and RKO cells were then transfected with a different lentivirus in 6-well cell culture plates and selected with 5μg/ml puromycin. For rescue experiments, the CRC cells and WASH5P-overexpressed cells were pretreated with 740Y-P (25 μM) for 24 h at 37°C.

### Cell Counting Kit-8 Analysis

Cell Counting Kit-8 (CCK-8) was used to detect cell viability. Cells were plated in 96-well plates at 5 × 10^3^ cells/well. Approximately 10 μl of CCK-8 solution was added to each well, and the color intensity with an enzyme-mark analyzer was measured after 0h, 24h, 48h, and 72 h, respectively, at 450 nm.

### Migration and Invasion Assay

The invasion assay was performed using 24-well Transwells (8-μm pore size; Millipore Bedford, MA, USA) that were coated with Matrigel (BD Biosciences, San Jose, CA, USA). Different cells were seeded in the serum-free medium into the upper chamber, whereas a medium supplemented with 20% FBS (fetal bovine serum) was applied to the lower chamber as a chemoattractant. After 48 h of incubation, the migrated cells at the bottom surface of the filter were fixed, stained, and counted.

The migration assay was performed using a 24-well transwell with no Matrigel. Treated cells were seeded in a serum-free medium into the upper chamber, whereas a medium supplemented with 20% fetal bovine serum was applied to the lower chamber as a chemoattractant. After 30 h of incubation, the migrated cells at the bottom surface of the filter were fixed, stained, and counted.

### Animal Experiments

5–6-week-old male Balb/c nude mice, weighing 18–20g, were purchased from Hunan SJA Laboratory Animal Co. Ltd. (Changsha, China) and housed in an SPF animal facility. All the mice were randomly assigned to the normal control (HCT116 vector) and lncRNA-WASH5P overexpression (HCT116 + WASH5P OE) group (n=6). The animal experiment was approved by the Ethics committee for animal experiments of the Affiliated Hospital of Qingdao University (No.QYFY WZLL 26861). The mice were housed 6/cage, in a 12:12-h light:dark cycle environment, with *ad libitum* access to food and water. After adapting to the new environment for 2 weeks, the mice were implanted with unilateral axilla subcutaneous injection of 1 × 10^7^ HCT116-vector cells or WASH5P-overexpressed HCT116 cells to induce primary tumors, respectively. The tumor length and width were measured every 5 days until the experiment was ended. After 4 weeks, the mice were sacrificed and the tumors were dissected and weighed.

In the metastatic model, all cells (1 × 10^6^) were transplanted by tail vein injection. All mice were sacrificed after 45 days. Lung tissues were collected for further analysis.

### Hematoxylin and Eosin Staining and Immunohistochemistry Analysis

Fresh tumor tissues from nude mice were fixed in formalin at room temperature for 24 h; then, the tissues were dehydrated with an ethanol and acetone series, embedded in paraffin, and sectioned onto glass slides at 4 μm. The slides were stained with hematoxylin and eosin (H&E) following deparaffinization. For IHC (immunohistochemistry), the sections were incubated at 65°C for 2 h and hydrated in xylene and graded ethanol. Then, the sections were immersed in a citrate buffer solution, heated in a microwave oven for 2.5 min, kept 5 min at room temperature, and heated in a microwave oven for 24 min. After washing with PBS (Phosphate Buffer Solution) three times, the slides were incubated with a blocking buffer for 10 min and then with a primary antibody overnight at 4°C. Primary antibodies include a rabbit antibody against vimentin (1:5,000; Proteintech, 10366-1-AP), the E-cadherin rabbit polyclonal antibody (1:1000; Proteintech, Wuhan, China, 20874-1-AP), p-AKT antibody (1:50; Y011054, abm, Richmond, BC, Canada), and AKT (1:200; Y409094, abm, Richmond, BC, Canada). After washing with Phosphate Buffer Solution, the sections were incubated with a secondary antibody, detected with DAB (diaminobenzidine), and counterstained with hematoxylin. Positive staining was deemed as brown dots.

### Statistical Analysis

R package clusterProfiler was used for GSEA (Gene Set Enrichment Analysis) analysis. The metrological data were expressed as means ± SD. Each experiment was performed at least 3 times. Significance was determined with the Student’s t-test (when only two groups were compared) or one-way analysis of variance (ANOVA) followed by Dunnett’s test (when more than two groups were compared). All statistical analyses were performed using SPSS (Statistical Product and Service Solutions) software (version 20.0), or GraphPad Prism 8.0 (La Jolla, CA, USA). P < 0.05 was considered statistically significant.

## Results

### Long Non-Coding RNA WASH5P is Downregulated in Colorectal Cancer

**Figure 1:**
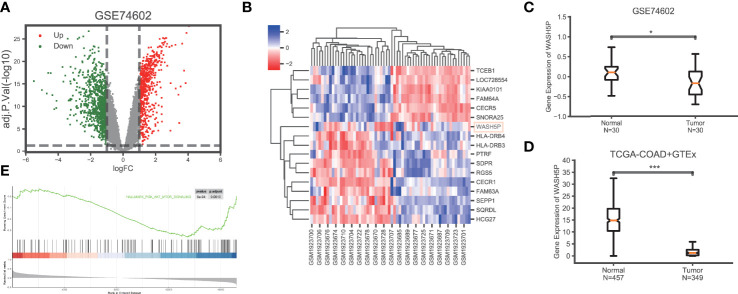
LncRNA WASH5P is downregulated in CRC. **(A)** Identification of the differentiated expression of non-coding genes in colorectal cancer based on GSE74602 from the The Cancer Genome Atlas (TCGA) database: a total of 1,639 differentially expressed non-coding genes were screened, including 662 upregulated (the red dots) and 977 downregulated genes (the green dots). **(B)** Heat maps showing the most differentiated expression genes between colorectal cancer (n=30) and adjacent normal tissues (n=30) from the TCGA data set (GSE74602). The color of the square corresponds to the relative expression among cases. **(C)** The relative expression of lncRNA WASH5P in CRC tissues (n=30) and adjacent non-tumor colorectal tissues (n=30) was analyzed in data set GSE74602. **(D)** WASH5P expression levels in cancer tissues (n=349) and normal tissues (n=457) in the database from TCGA-COAD and GTEx.***. Correlation is significant at the 0.001 level. **(E)** GSEA analysis showed that lncRNA WASH5P was enriched in the hallmark PI3K-AKT pathway.

To explore the key lncRNAs in CRC carcinogenesis, we analyzed the gene expression data in CRC (n=30) and paired adjacent tissues (n=30) in GSE74602 from the TCGA database. A total of 1,639 non-coding genes were differentially expressed, including 662 upregulated and 977 downregulated genes in CRC compared with the paired adjacent tissues (**Figure 1A**). The non-coding genes with the most significant differential expression in GSE74602 are listed in **Figure 1B**. Among these genes, lncRNA WASH5P was significantly downregulated in CRC tissues compared with adjacent tissues using the Wilcoxon rank-sum test in GSE74602 (logFC= -1.09, adj. P=0.0227) (**Figure 1C**). Summarized data from TCGA-COAD (The Cancer Genome Atlas-Colon Adenocarcinoma) and GTEx (the Genotype-Tissue Expression) validated that the expression level of WASH5P was significantly lower in cancer tissues than in normal tissues (P<0.001) (**Figure 1D**). Furthermore, Gene Set Enrichment Analysis analysis showed that lncRNA WASH5P was enriched in the hallmark PI3K-AKT pathway (**Figure 1E**).

**Figure 2:**
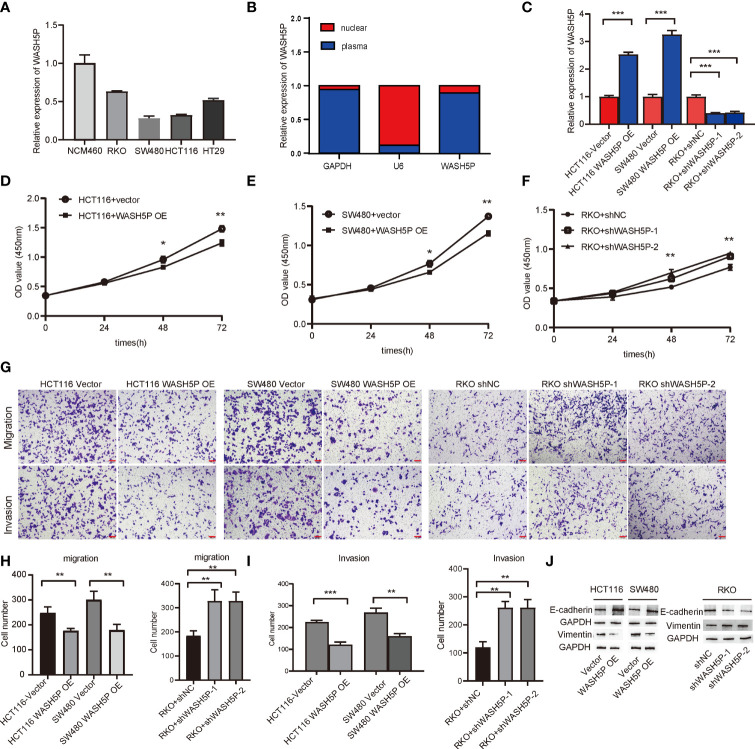
LncRNA WASH5P inhibits the development of CRC *in vitro*. **(A)** LncRNA WASH5P expression was measured in the normal colon cell line NCM460 and four CRC cell lines by real-time PCR. **(B)** Real-time PCR was performed to examine lncRNA WASH5P levels in the cytoplasm and nucleus, respectively. **(C)** Knockdown and overexpression efficiency were confirmed by real-time PCR in different CRC cell lines. ***. Correlation is significant at the 0.001 level. **(D, E)** CCK-8 assay was performed in HCT116 and SW480 cells transfected with lncRNA WASH5P or a vector. Cell viability was measured from an absorbance reading at 450 nm. Data were expressed as relative viability (%) calculated: [A450(treated) − A450 (blank)]. *. Correlation is significant at the 0.05 level, **. Correlation is significant at the 0.01 level. **(F)** CCK-8 assay was performed in RKO cells transfected with shWASH5P (WASH5P shRNA) or shNC (control shRNA). **. Correlation is significant at the 0.01 level. **(G–I)** *In vitro* invasion and migration assays were performed using HCT116, SW480, and RKO cells. Cells were photographed under a light microscope and counted from five random microscopic fields (×200) per insert in triplicate. **. Correlation is significant at the 0.01 level, ***. Correlation is significant at the 0.001 level. Scale bars represent 100 µm. **(J)** Protein level of epithelial marker (E-cadherin) and mesenchymal marker (vimentin) were measured by Western blot in HCT116 and SW480 cell after WASH5P transfection, compared with vector; Immunoblots indicated the expression levels of epithelial (E-cadherin) and mesenchymal (vimentin) markers in RKO cells after being transfected with shWASH5P and shNC. GAPDH was used as a loading control.

To verify the expression level of lncRNA WASH5P, qPCR was performed in different CRC cell lines (SW480、HCT116、RKO、HT29) and one normal colon cell line (NCM460). The results showed that lncRNA WASH5P was significantly downregulated in CRC cell lines compared with normal colon cells (p<0.05) (**Figure 2A**). To figure out the location of lncRNA WASH5P in CRC cells, we analyzed the expression level of lncRNA WASH5P both in the nucleus and the cytoplasm and found that lncRNA WASH5P was mainly located in the cytoplasm (**Figure 2B**).

### WASH5P Inhibits Colorectal Cancer Cell Proliferation, Invasion, and Migration

We then evaluated the role of lncRNA WASH5P in CRC cells. LncRNA WASH5P was successfully downregulated or upregulated in different CRC cell lines (**Figure 2C**). The overexpression of lncRNA WASH5P in HCT116 and SW480 could dramatically inhibit tumor cell viability (**Figures 2D, E**). The transwell assay revealed that lncRNA WASH5P upregulation could significantly inhibit tumor migration and invasion when compared with the control group (**Figures 2G–I**). Furthermore, the silence of lncRNA WASH5P could promote RKO CRC cell viability (**Figure 2F**), migration, and invasion (**Figures 2G–I**). In addition, the overexpression of WASH5P could inhibit the EMT process *via* targeting EMT markers, while the downregulation of WASH5P may promote EMT as shown in **Figure 2J**.

### WASH5P Plays an Essential Role in Inhibiting the Development of CRC Through the AKT Pathway

**Figure 3:**
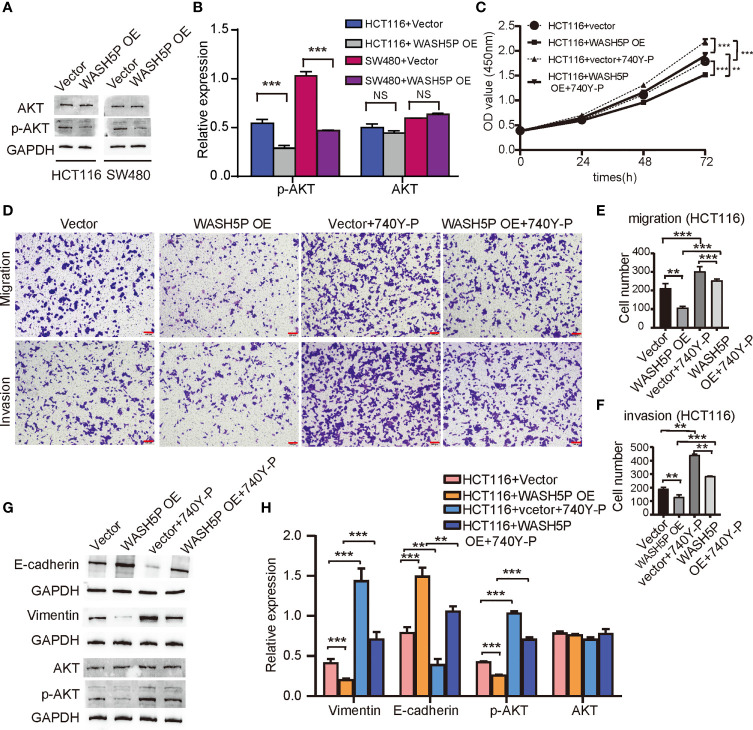
LncRNA WASH5P plays an essential role in inhibiting the development of CRC through the AKT pathway. **(A, B)** Western blot analysis and summarized data showed that the expression of p-AKT was downregulated in HCT116 and SW480 cells transfected with lncRNA WASH5P compared with a vector. ***. Correlation is significant at the 0.001 level. **(C)** CCK-8 assay was performed in HCT116 cells transfected with lncRNA WASH5P or vector. 740Y-P was added to the medium and cultured for 24 h. Cell viability was measured from an absorbance reading at 450 nm. Data were expressed as relative viability (%) calculated: [A450(treated)-A450 (blank)]. **. Correlation is significant at the 0.01 level, ***. Correlation is significant at the 0.001 level. **(D–F)** Transwell Matrigel invasion assays and Migration assays were performed in HCT116 cells transfected with different viruses as indicated. Cells were observed under a light microscope and photographed. Cells were counted from five random microscopic fields (×200) per insert in triplicate. The migrated cell numbers were normalized to that of the control group. Data are shown as mean ± SD from three separate experiments. **. Correlation is significant at the 0.01 level, ***. Correlation is significant at the 0.001 level. Scale bars represent 100 µm. **(G, H)** Immunoblots and summarized data showed the expression of AKT, p-AKT, epithelial marker (E-cadherin), and mesenchymal marker (vimentin) in shWASH5P or shNC transfected HCT116 cells after 740Y-P treatment. GAPDH was used as a loading control. Data are shown as mean ± SD from three separate experiments. **. Correlation is significant at the 0.01 level, ***. Correlation is significant at the 0.001 level.

We have found that lncRNA WASH5P was enriched in the hallmark PI3K-AKT pathway in Gene Set Enrichment Analysis analysis (**Figure 1E**). PI3K-AKT is a classical signaling pathway that is involved in CRC tumorigenesis, so we further determined the relationship and mechanisms involved in WASH5P and CRC. Western blot and summarized data suggested that the overexpression of WASH5P in HCT116 and SW480 could significantly inhibit AKT pathway activation *via* suppressing AKT phosphorylation (**Figures 3A, B**). Therefore, we speculate that the AKT pathway might be a potential downstream regulator of WASH5P in CRC tumorigenesis. In order to confirm this, we then treated the WASH5P-overexpressed cells and the control group with 740Y-P, a specific activator of PI3K, to activate the PI3K/AKT pathway. Our data suggested that 740Y-P treatment could restore WASH5P-reduced CRC cell viability in WASH5P-overexpressed cells (**Figure 3C**). Moreover, 740Y-P abolished the inhibitory effects of WASH5P on cell migration and invasion in WASH5P-overexpressed cells *via* the cell migration assay and cell invasion assay (**Figures 3D–F**). In addition, 740Y-P could rescue the WASH5P-induced downregulation of p-AKT and vimentin and the upregulation of E-cadherin *via* Western blot (**Figures 3G, H** **)**.

All these results demonstrated that WASH5P could inhibit the development of CRC *via* the AKT signal pathway.

### WASH5P Significantly Inhibits CRC Carcinogenesis *In Vivo*

**Figure 4:**
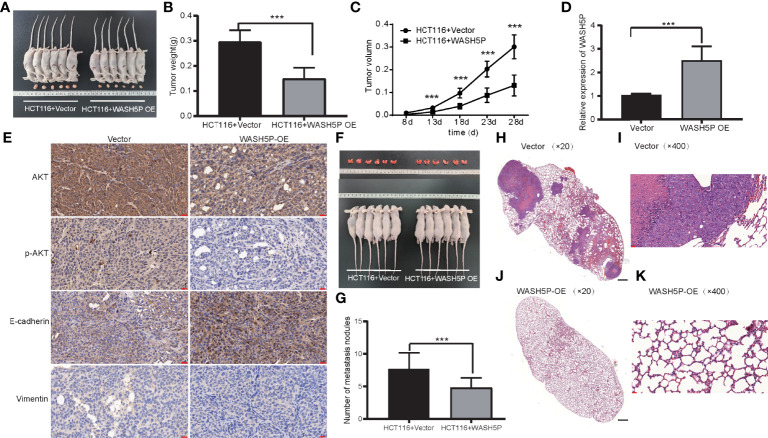
LncRNA WASH5P significantly inhibits CRC *in vivo*. **(A)** Mice carrying xenograft tumors treated with lncRNA WASH5P (n=6, right panel) or vector (n=6, left panel) at the last time point. **(B)** The xenograft tumors were separated and weighted at the end of the experiments. ***. Correlation is significant at the 0.001 level. **(C)** Tumor volume was measured in lncRNA WASH5P or vector groups. Data represent the mean with a 95% confidence interval. ***. Correlation is significant at the 0.001 level. **(D)** Expression level of WASH5P was confirmed by real-time PCR in the xenograft tumors of the WASH5P-overexpressed group and vector group. ***. Correlation is significant at the 0.001 level. **(E)** Immunohistochemical analysis revealed the expression of AKT, pAKT, epithelial (E-cadherin), and mesenchymal (vimentin) markers in the tumors of different groups. Original magnification: ×200. Scale bars represent 20 µm. **(F)** Pulmonary images of mice in a metastatic mouse model treated with vector (n=6, left panel) or lncRNA WASH5P (n=6, right panel) at the last time point. **(G)** Summarized data of tumor lung nodules in nude mice at 45 days in lncRNA WASH5P or vector groups; n = 6. ***. Correlation is significant at the 0.001 level. **(H–K)** Representative H&E images of the lungs of nude mice metastasis at 45 days in two groups: vector group **(H, I)** and WASH5P-overexpressed group **(J, K)**. The black scale bars in H and J represent 500 µm. The red scale bars in I and K represent 20 µm.

To further identify the involvement of WASH5P *in vivo*, we established the xenograft tumor model. WASH5P overexpression could inhibit tumor growth in the mouse model when compared with the control group (**Figures 4A–C**). Furthermore, we analyzed the expression of vimentin, E-cadherin, p-AKT, and AKT in the tumor mass and found that vimentin and p-AKT were decreased in the WASH5P-overexpressed group compared to normal control (**Figures 4D, E**). These data suggested that the overexpression of WASH5P inhibited CRC progression *via* regulating the AKT pathway *in vivo*.

In addition, we evaluated the effect of WASH5P on CRC in the metastatic mouse model. WASH5P overexpression could significantly inhibit CRC lung metastasis when compared with the control group *via* H&E staining (**Figures 4F–K**), indicating that WASH5P could inhibit CRC metastasis *in vivo*.

## Discussion

Emerging pieces of evidence showed that lncRNAs could function as important modulators in carcinogenesis, and emerge as oncogenes or tumor suppressors, *via* target genes or signaling pathways (9, 14, 16). Specific lncRNAs in clinical samples could serve as novel biomarkers or therapeutic targets and contribute to tumor diagnosis and better clinical outcomes (17–20).

To figure out the potential lncRNAs in CRC, we performed bioinformatics analysis based on the TCGA database, and the results showed that WASH5P was dramatically downregulated in CRC. Right now, no studies have been reported about the role of lncRNA WASH5P in any disease.

LncRNA WASH5P is one of the newest lncRNAs with a length of 400 bp and is mainly located in the cytoplasm. In our present study, we showed that lncRNA-WASH5P regulated the proliferation, migration, and invasion process of CRC. Firstly, WASH5P was downregulated in CRC tissues compared to adjacent controls. Secondly, WASH5P was decreased in four CRC cell lines. Thirdly, the silence of WASH5P could aggregate CRC cell proliferation, invasion, and migration *via* the AKT pathway. Additionally, the overexpression of WASH5P inhibits this phenomenon. Finally, the overexpression of WASH5P in HCT116 could inhibit CRC proliferation and invasion *in vivo*. All the above results indicated the therapeutic potential of WASH5P in CRC.

The pivotal role of the PI3K/AKT pathway in carcinogenesis has received much attention (21, 22). The PI3K/AKT pathway could participate in cell proliferation, differentiation, angiogenesis, and invasion in CRC (23, 24). More and more sheds of evidence have emphasized that lncRNAs could promote CRC carcinogenesis *via* activating the PI3K/AKT pathway (16, 25, 26). In the present study, Gene Set Enrichment Analysis analysis showed that lncRNA WASH5P was enriched in the hallmark PI3K-AKT pathway. The overexpression of WASH5P in CRC cells could significantly inhibit AKT pathway activation. The further functional experiment revealed that 740Y-P could restore the expression of p-AKT and promote CRC in WASH5P-overexpressed cells. Thus, our result provided evidence that WASH5P could inhibit CRC *via* targeting AKT signaling, indicating the possible mechanism of this potential biomarker.

Interestingly, emerging pieces of evidence showed that lncRNAs could be used as drugs to treat cancer due to their biological functions (18, 27). For example, lncRNA MEG3 was combined with exosome through engineering technology and used to treat osteosarcoma animal models (28). Furthermore, pieces of evidence were provided in clinical studies. A phase I/II clinical trial showed that BC-819, a plasmid with an lncRNA H19 promoter, showed 22% complete response rates and 44% partial response rates in treating bladder cancer (29). Antisense oligonucleotides (ASOs) containing lncRNAs were shown to be successful in treating neurodegenerative diseases both in preclinical studies and human clinical trials (30). In addition, the clinical trials of ASO- targeting lncRNAs have shown a promising therapeutic effect in advanced unresectable solid tumors (NCT02508441 (31)/NCT03985072 (32)).

**Figure 5:**
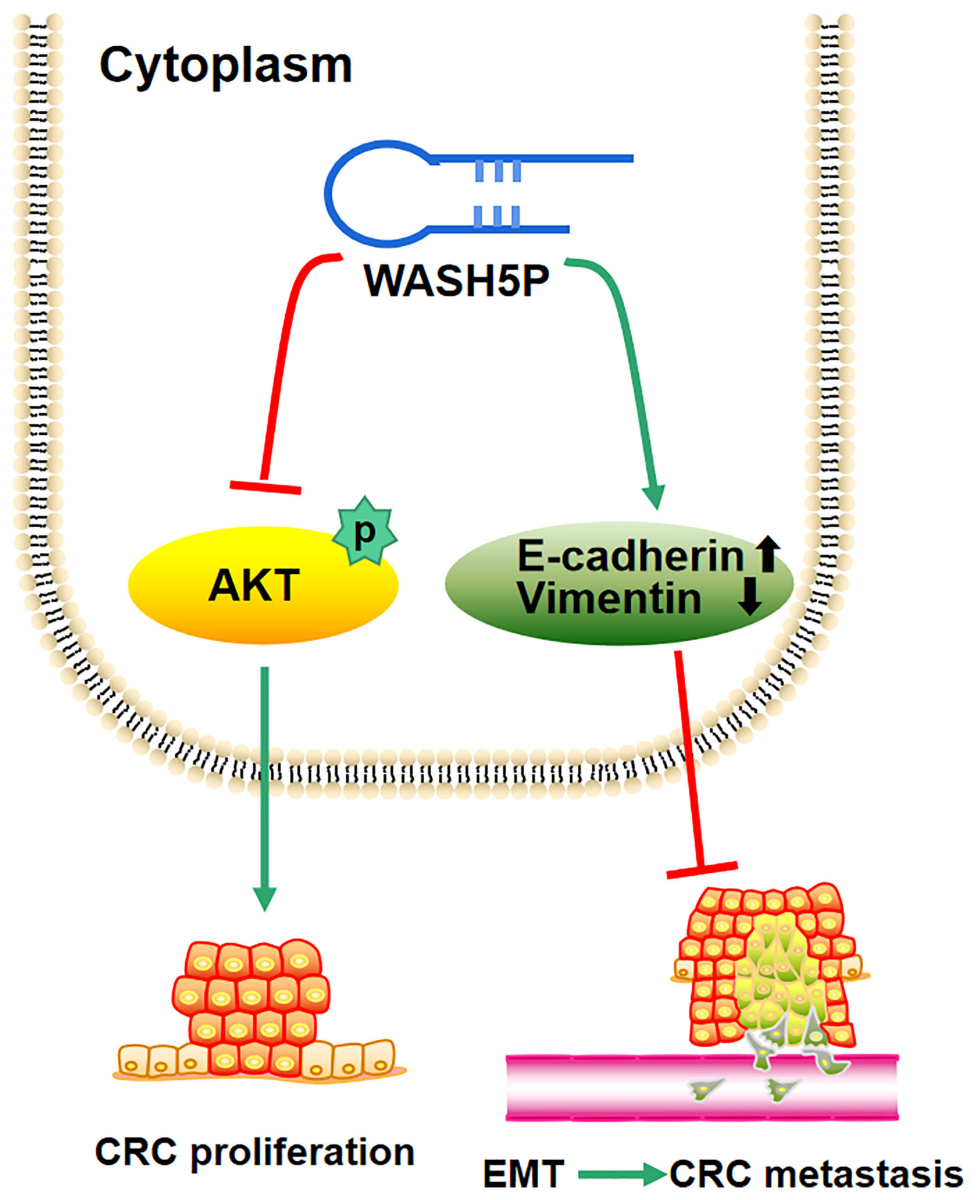
Schematic model of the role of lncRNA WASH5P in colorectal cancer. lncRNA WASH5P could inhibit CRC *via* repressing the AKT signaling pathway and EMT.

In summary, the present study supplies the new evidence that the tumor suppressor WASH5P could inhibit CRC cell proliferation, invasion, and migration through the AKT signaling pathway both *in vitro* and *in vivo* (**Figure 5**). Therefore, the detection and targeting of WASH5P could monitor and suppress CRC development, providing a new potential biomarker and therapeutic target for CRC.

## Data Availability Statement

The original contributions presented in the study are included in the article/supplementary material. Further inquiries can be directed to the corresponding author.

## Ethics Statement

The animal study was reviewed and approved by the Ethics committee for animal experiments of the affiliated hospital of Qingdao university.

## Author Contributions

HW, ZT, and LR designed this research. HW, TM, and QZ performed the analyses and prepared the manuscript. HW, KR, YZ, and XQ performed literature search and the initial analyses. HW, BC, TM, YJ, and LR performed analyses and data interpretation. HW and LR edited the manuscript. All authors have read and agreed to the published version of the manuscript.

## Funding

This work was supported by the National natural science foundation of China (NO. 81602056) to LR, the Natural Science Foundation of Shandong Province (NO. ZR2016HQ45, ZR2020LZL004) to LR, Shandong medical and health science and technology development plan project (Grant number 202003030357), and Postdoctoral Science Foundation of China (RZ2100002858) to HW.

## Conflict of Interest

The authors declare that the research was conducted in the absence of any commercial or financial relationships that could be construed as a potential conflict of interest.

## Publisher’s Note

All claims expressed in this article are solely those of the authors and do not necessarily represent those of their affiliated organizations, or those of the publisher, the editors and the reviewers. Any product that may be evaluated in this article, or claim that may be made by its manufacturer, is not guaranteed or endorsed by the publisher.
